# The Fish-Specific Protein Kinase (PKZ) Initiates Innate Immune Responses via IRF3- and ISGF3-Like Mediated Pathways

**DOI:** 10.3389/fimmu.2019.00582

**Published:** 2019-03-28

**Authors:** Xiaowen Xu, Meifeng Li, Chuxin Wu, Dongming Li, Zeyin Jiang, Changxin Liu, Bo Cheng, Huiling Mao, Chengyu Hu

**Affiliations:** ^1^College of Life Science, Nanchang University, Nanchang, China; ^2^Fuzhou Medical College, Nanchang University, Fuzhou, China

**Keywords:** PKZ, PRR, IRF3-mediated and ISGF3-like mediated pathways, innate immunity, teleost

## Abstract

PKZ is a fish-specific protein kinase containing Zα domains. PKZ is known to induce apoptosis through phosphorylating eukaryotic initiation factor 2α kinase (eIF2α) in the same way as double-stranded RNA-dependent protein kinase (PKR), but its exact role in detecting pathogens remains to be fully elucidated. Herein, we have found that PKZ acts as a fish-specific DNA sensor by initiating IFN expression through IRF3- or ISGF3-like mediated pathways. The expression pattern of *PKZ* is similar to those of innate immunity mediators stimulated by poly (dA:dT) and poly (dG:dC). DNA-PKZ interaction can enhance PKZ phosphorylation and dimerization *in vitro*. These findings indicate that PKZ participates in cytoplasmic DNA-mediated signaling. Subcellular localization assays have also shown that PKZ is located in the cytoplasm, which suggests that PKZ acts as a cytoplasmic PRR. Meanwhile, co-IP assays have shown that PKZ can separately interact with IRF3, STING, ZDHHC1, eIF2α, IRF9, and STAT2. Further investigations have revealed that PKZ can activate IRF3 and STAT2; and that IRF3-dependent and ISGF3-like dependent mediators are critical for PKZ-induced IFN expression. These results demonstrate that PKZ acts as a special DNA pattern-recognition receptor, and that PKZ can trigger immune responses through IRF3-mediated or ISGF3-like mediated pathways in fish.

## Highlights

- The expression pattern of *PKZ* is similar to those of IRF3-dependent and ISGF3-like-dependent mediators.
- *In vitro*, PKZ can be activated by poly (dA:dT)- or poly (dG:dC)-PKZ interaction, but PKZ cannot interact with RNA.
- PKZ activates IFN expression through IRF3- or ISGF3-like-dependent pathway.

## Introduction

Innate immunity is the first defense line for vertebrates, especially lower vertebrates, to fight off invading pathogens (1, 2). Pattern-recognition receptors (PRRs) that recognize pathogen-associated molecular patterns (PAMPs) play vital roles in initiating IFN signaling pathways (3–5). In recent years, many complicated and effective PRRs have been identified in mammals. A few toll-like-receptors (TLR) and RIG-I-like receptors (RLR) are known to detect bacterial and viral RNAs (6, 7). TLR3 recognizes double-stranded RNA (dsRNA), and then triggers IFN-β expression by activating interferon transcription factor 3 (IRF3) and nuclear factor κB (NF-κB) (8, 9). RLRs (RIG-I and MDA5) detect cytosolic RNA via the adaptor protein IPS-1 and activate IFN-β signaling pathways (10).

In addition to the RNA sensors described above, a number of intracellular DNA sensors have been confirmed in recent times, such as absent in melanoma 2 (AIM2) (11), DNA-dependent activator of IFN-regulatory factors (DAI) (12), IFN-inducible protein 16 (IFI16) (13), DEAD (Asp-Glu-Ala-Asp) box polypeptide 41 (DDX41) (14), RNA polymerase III (15), and Cyclic GMP-AMP synthase (cGAS) (16). These proteins are important in responding to various DNA infections. For example, IFI16 is a sensor for HIV DNA through interaction with stimulator of IFN gene (STING), and is also important in inducing IFN-β reactions in human macrophages (13). Cyclic GMP-AMP synthase (cGAS) is known to detect cytosolic DNA in various cell types (16). Recognition of viral DNA by cGAS induces the synthesis first of the second message cGAMP from ATP and GTP, and then of cGAMP which binds to STING; and STING enhances IRF3 phosphorylation by TBK1, thus activating downstream genes (17–21).

In recent years, much progress has been made in the study of fish PRRs. Along with the discovery of RNA sensors in mammals, RLRs (RIG-I, MDA5, and LGP2) and TLRs, which are involved in dsRNA recognition, were also identified in teleost fish (22–27). While the findings on RNA-recognizing PRRs in fish are quite clear, those on DNA-recognizing PRRs are ambiguous. A novel member of vertebrate eIF2α kinase, named PKZ (protein kinase containing Zα binding domains), has been identified exclusively in fish (28–30). The Zα binding domains of fish PKZ similar to the DNA sensors in mammals (28–30). This suggests PKZ might be a special DNA sensor in fish (31). Recent studies have demonstrated that fish PKZ can specifically respond to poly (dG:dC), then phosphorylate eIF2α, and finally lead to apoptosis (30, 32, 33).

The present study has demonstrated that the expression profile of *PKZ* is similar to those of IFN response mediators; that PKZ can specifically recognize poly (dA:dT) and poly (dG:dC) but not poly I:C (dsRNA analog) *in vitro*; and that PKZ is located in the cytoplasm and is a basic factor in DNA recognition. As well, when cells are stimulated by invading DNA molecules, PKZ can interact with and activate the mediators in the IRF3-dependent or ISGF3-like dependent pathway. Our findings suggest that PKZ performs as a special DNA-sensing receptor and triggers immune responses through the IRF3-dependent or ISGF3-like dependent pathway.

## Materials and Methods

### Cells and Virus Analogs

Grass carp (*Ctenopharyngodon idellus*) ovary cells (CO) and *C. idellus* kidney cells (CIK) were cultured in medium 199 supplemented with 10% FCS at 28°C. Human Embryonic Kidney 293T cells (HEK-293T) were maintained at 37°C under 5.0% CO_2_ in DMEM supplemented with 10% FCS. CO cells are superior to CIK cells due to its stronger ability of expression and better cell morphology. In addition, HEK-293T cells were used instead of CIK and CO cells in immunoprecipitation assay because of their super-high transfection efficiency.

ISD-PS (biotin tagged ISD-PS), poly (dA:dT) [biotin tagged poly (dA:dT)] and poly (dG:dC) [biotin tagged poly (dG:dC)] were all synthesized by Sangon Biotech (China) and their sequences were presented in Table 1. ISD-PS is an RNA analog carrying a biotin tag (34). Poly I:C was purchased from Sigma (USA). The nucleic acids [poly (dA:dT), poly (dG:dC), and ISD-PS] were synthesized in accordance with the manufacturer's protocols from 10 μl DNA oligo, 2 μl 10 × oligo annealing buffer (100 mM Tris-HCl, pH 8.0, 10 mM EDTA, pH8.0, 1 M NaCl), and 8 μl DNase/RNase-free water. The 20 μl total- reaction mixture was incubated at 95°C for 4 min, and then kept at room temperature for 5–10 min.

**Table 1:** The nucleotide sequence of primers in this study.

**Primer name**	**Primer sequence (5^**′**^-3^**′**^)**	**Application**
PKZ-RT-F	CACCGTGAACAGACATTTG	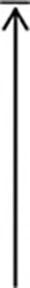
PKZ-RT-R	TCCCTTACGTGTTTCTCTTC	
STING-RT-F	ACATAGCAGGGGTACGGAAT	
STING-RT-R	CCATGTGAATCTCTCCGTCC	
ZDHHC1-RT-F	GTCTGTCACCTCTTCATGCA	
ZDHHC1-RT-R	CAGGAGAGCAGAAATCACAC	
IFN-RT-F	GTCAATGCTCTGCTTGCGAAT	Real-time PCR
IFN-RT-R	CAAGAAACTTCACCTGGTCCT	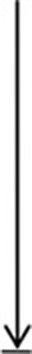
TBK1-RT-F	GAGACATCAAGCCAGGGAAC	
TBK1-RT-R	AAAACGTGACTCCGATGCTC	
IPS-1-RT-F	AATTTGGCTCGCTTTTCGTCA	
IPS1-RT-R	TCATCAGCCAGTTCCCTATGT	
IRF3-RT-F	TCCAGGCCAAGCATACGAA	
IRF3-RT-R	CCATTTGCAACAGCCATCAT	
IRF3-ORF-F	ATGACCCATCCAAAACCGCT	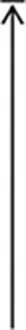
IRF3-ORF-R	TCACTTGGTGTCACACAACTC	
ZDHHC1-ORF-F	ATGGACGTCTGCAGTAAGAAC	
ZDHHC1-ORF-R	TGTGGTGCTGGTGTCAATG	
STING-ORF-F	AATGTGTGGTGTGATCGGAG	
STING-ORF-R	CGAATAATCAGTAGTCTCCTCAGG	
ADAR1-ORF-F	ATGAGCAGAGGTAGAGGAGGGTTT	
ADAR1-ORF-R	CTAAAAGCCCTGTGCTGCAGA	
PKZ-ORF-F	ATGTCTGCCGAAACTCAAATG	Eukaryotic expression vector construction
PKZ-ORF-R	TCAAATCGTTTTCTGGCTTAACA	
eIF2α-ORF-F	ATGCCCGGACTCAGCTGTA	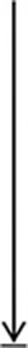
eIF2α-ORF-R	AGCTAGTCATCAGTCTTGGCCTC	
IRF9-ORF-F	ATGGCATCTGGAAGGATTCGT	
IRF9-ORF-R	GGGCTTTAATGTCAAGAATGCAGC	
STAT1-ORF-F	ATGGCACTTTGGAACCAGCTGC	
STAT1-ORF-R	ACACTTTTGGAGTCTTGAAACAGGG	
STAT2-ORF-F	AGAATGACTCAGTGGGACCG	
STAT2-ORF-R	AGGGGTCATGGATCAAATTCAGG	
STAT6-ORF-F	ACAACAAACGCACACGGAA	
STAT6-ORF-R	GACCGTACCTCAAAATGATGACAA	
Poly (dA: dT)	CTGATACTACATTGAATTCTATATATA TATATATATATAGAATTCAAT GTAGTATCAGA	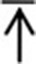
		DNA analog
Poly (dG: dC)	CTGATACTACATTGAATTCGCGCGC GCGCGCGCGCGCGCGAATTC AATGTAGTATCAGA	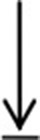
ISD-PS	TACAGATCTACTAGTGATCTATGAC TGATCTGTACATGATCTACA	RNA analog
siRNA-PKZ	GCCAGACUAGACCAUCCAATT	siRNA

### Plasmids and Recombinant Construction

pCMV-FLAG and pcDNA3.1(+) were both purchased from Invitrogen (USA). pEGFP-C1, pGL3, and *E. coli* strains DH5α were all bought from Promega (USA). The *C. idellus* open reading frames (ORFs) of *PKZ, IRF3, STING, ZDHHC1, IRF9, STAT2, eIF2*α, and *ADAR1* were prepared, and each of them was separately inserted into pCMV-FLAG, pEGFP-C1, and pcDNA3.1(+) vectors to construct expression plasmids. They were used for *in vitro* translation, nucleic acids pull down, sub-localization, and co-IP assays. Each of the *C. idellus* mutants of *PKZ-C* (176–513 aa), *PKZ-N* (1–169 aa), and *PKZ-198K (K198R)* was separately inserted into pcDNA3.1 (+); meanwhile, each of the mutants mentioned above was also individually inserted into pCMV-FLAG (Figure 3A). All of the constructs were confirmed by DNA sequencing. The primers used for plasmid construction were given in Table 1. The plasmids, genes, types of experiment, and Genbank ID for this paper were listed in Table 2.

**Table 2:** The plasmids, genes, types of experiment, and genebank ID were used in the article.

**Plasmids**	**Genes**	**Types of experiment**	**GeneBank ID**	
pcDNA3.1	PKZ, IRF3, STING, ZDHHC1, IRF9, STAT2,	qRT-PCR, Western blot, Dual-luciferase assay, RNAi	PKZ	GU299765.1
			PKR	JX511974.1
			IRF3	KC898261.1
pCMV-FLA	PKZ, IRF3	Western blot, DNA-pulldown, Immunoprecipitation assay	STING	KF494194.1
			ZDHHC1	KY680346.1
			IRF9	KT601055.1
pEGFP-C1	PKZ, ADAR1, IRF3, STING, ZDHHC1, IRF9, STAT1, STAT2, STAT6, eIF2α	Subcellular localization, Immunoprecipitation assay	STAT1	KU508677.1
			STAT2	KT781914.1
			STAT6	MG384738.1
			eIF2α	KX906957
			IFN	DQ357216.1
pGL3	IFN	Dual-luciferase assay	ADAR1	KU198336.1
			IPS-1	KF366908.1
			TBK1	JN704345.1

### Luciferase Activity and Quantitative RT-PCR Assays

Each plasmid was transfected according to the FuGENE®6 (Promega, USA) protocol. Each well of 6-well plate was transfected with 2 μg of plasmid, and each 10-cm dish was transfected with 5 μg of plasmid. In nucleic acid stimulation, nucleic acid (2 μg) was transfected into cells. In luciferase activity assays, 0.25 μg of *PKZ*-pcDNA3.1 (or basic-pcDNA3.1 plasmid), 0.25 μg of *IFN-pro*-pGL3, and 0.025 μg of pRL-TK Renilla luciferase vectors were transfected in CIK or CO cells. IFN promoter was inserted into pGL3 plasmid. As this recombinant plasmid was used in dual-luciferase activity assay, the luciferase activity of the promoter can reflect *IFN* transcription levels.

In the quantitative RT-PCR assay, CIK cells were seeded in 6-well plates and transfected with 2 μg of poly (dA:dT) or 2 μg of poly (dG:dC). After all RNA was extracted from CIK cells, qRT-PCR was also used to detect the expression levels of *IFN, IPS-1, TBK1*, and *PKZ*. Grass carp *IFN* expression was detected in CO cells transfected with 2 μg of *PKZ*-pcDNA3.1, 2 μg of *IRF3*-pcDNA3.1, 2 μg of *STING*-pcDNA3.1, 2 μg of *ZDHHC1*-pcDNA3.1, 2 μg of *IRF9*-pcDNA3.1, 2 μg of *STAT2*-pcDNA3.1, and 2 μg of empty vectors. Twenty-four hours later, the cells were transfected with 2 μg of poly (dG:dC) for 6 h.

### Subcellular Localization Analysis

In subcellular localization analysis, CO cells were plated on 35 cm^2^ microscopic petri dishes. After 12 h they were transfected with *PKZ*-GFP, *ADAR1*-GFP, and GFP-C1. Twenty-four hours post transfection, the cells were washed three times with PBS, and fixed with 4% (v/v) paraformaldehyde at room temperature for 15 min. The cells were then dyed with DAPI (0.1 μg/ml) and examined under a confocal microscope (Olympus, FV1000).

### Nucleic Acids Pulldown Assays

In nucleic acids pulldown assays, synthetic ISD-PS was used as RNA analog. Two microgram each of biotin-labeled ISD-PS, poly (dA:dT), and poly (dG:dC) were incubated with M-280 streptavidin-coupled Dynabeads (Invitrogen) for 5 h. The unbound biotin-labeled nucleic acids were washed with 1 × BW buffer (5 mM Tris-HCl pH 7.5, 0.5 mM EDTA, 1 M NaCl) five times and immobilized on M-280 streptavidin-coupled Dynabeads (Invitrogen) at 4°C. CO cells were plated on 10 cm diameter dishes and transfected with 5 μg of *PKZ*-FLAG (or *PKZ-C*-FLAG or *PKZ-N*-FLAG) plasmids. Twenty-four hours later, the cells were stimulated with poly (dG:dC) for 12 h. The medium was carefully removed and washed twice with PBS, and lysates were cleared by sonication and centrifugation. Cleared lysates were incubated with Dynabeads which were separately immobilized with biotin-nucleic acids (poly (dA:dT), poly (dG:dC) and ISD-PS at 4°C on a rotary wheel for 12 h. The experiment was divided in two groups. In non-competitor group, lysates were incubated with Dynabeads immobilized with biotin-nucleic acids; in competitor group, lysates, and competitors were incubated with Dynabeads immobilized with biotin-nucleic acids. Ten micrograms each of non-biotin tagged nucleic acids (poly (dA:dT), poly (dG:dC), and ISD-PS) acted as competitors. Highly concentrated non-biotin tagged nucleic acids in incubations (lysates and Dynabeads immobilized with biotin-nucleic acids) will competitively interact with proteins. The unbound proteins in Dynabeads were removed by five consecutive washes with 1 × BW buffer. Given that the proteins can interact with nucleic acids, PKZ-FLAG can be detected in the non-competitor group and cannot be detected in the competitor group. The beads of what were eluted with 100 μl of 2 × SDS sample buffer by boiling for 7 min at 95°C, before performing Western blot analysis.

### Antibodies, Western Blot, Co-immunoprecipitation (co-IP) Assays, and Analysis of Phosphorylation State

Mouse monoclonal antibodies against FLAG and GFP were purchased from Sigma (USA) and Abmart (USA), respectively. Phospho-IRF3 (Ser 396) rabbit monoclonal antibody was purchased from Cell Signaling Technology (USA). Anti-GAPDH rabbit polyclonal antibody was purchased from CWBIO (China). Rabbit polyclonal anti-grass carp PKZ, IFN, IRF3, STING, and ZDHHC1 antisera were prepared in our lab (33, 35). HEK-293T cells were used in co-IP assays instead of fish cells because of their superior transfection efficiency. Co-IP assays and Western blot were performed as previously described (36, 37).

In the absence of specific phospho-PKZ and phospho-STAT2 antibodies, immunoprecipitation assays were performed to study PKZ and STAT2 phosphorylation. To begin with, CO cells were transfected with 2 μg of *PKZ*-FLAG/*STAT2*-GFP plasmids. Twenty-four hours later, the cells were separately stimulated with poly I:C, poly (dA:dT) and poly (dG:dC) for 12 h. All the proteins were then harvested with lysis (Transgene, China). First, the amount of PKZ-FLAG/STAT2-GFP proteins was determined by Western blot. The concentrations of protein in lysates were determined by an Enhanced BCA Protein Assay kit (Beyotime). Then the same amount of PKZ-FLAG/STAT2-GFP was immunoprecipitated from cell lysates. Finally, the phosphorylation of PKZ/STAT2 was detected with an anti-phospho-Ser/Thr/Tyr antibody (AnaSpec, CA).

### Native PAGE

CO cells were separately transfected with the negative control (4 μg of basic-pcDNA3.1), the positive control [4 μg of poly I:C, 4 μg of poly (dA:dT), 4 μg of poly (dG:dC)], the experimental group (4 μg of *PKZ*-pcDNA3.1) plasmids. The lysis of total protein was studied in two groups of experiment. One group was detected in SDS-PAGE; another group detected in native PAGE. Native PAGE assays were conducted with 8% acrylamide gel without SDS. In the same way as described previously, the sample was dissolved in 5 × loading buffer without SDS. The gel was run with 0.5% deoxycholate, 25 mM Tris-HCl (pH 8.4), and 192 mM glycine for 60 min at 35 mA on ice, and then immunoblotted to a nitrocellulose membrane (Millipore, USA).

### RNA Interference-Mediated Gene Knockdown Assays

In the RNA interference-mediated gene knockdown assays, the specific siRNA sequence against *C. idellus PKZ, IRF9*, and the negative control RNA (*N.C*.) oligonucleotides were prepared (Shanghai GenePharma Co., Ltd.) (Table 1). The siRNA sequence against *IRF3* was used as in the previous study (35). The transfection reagent, Hiperfect® (Qiagen, Germany), has been widely used in our previous study (35). The transfection was carried out according to the manufacturer's protocol and instructions.

### Data Analysis

Statistical analysis of qRT-PCR was performed and graphs were prepared using Microsoft excel. The Gray value of western blot was analyzed and confocal images were made by image J. The diagram of signaling pathway was constructed with the Portable Pathway Builder Tool 2.0. Data analysis of qRT-PCR and dual-luciferase assay were both presented using the unpaired two-tailed *t*-test, and *p* < 0.05 were considered statistically significant (^*^p < 0.05, ^**^p < 0.01).

## Results

### PKZ and Some Mediators of Immune Responses Are Up-Regulated Under Stimulation With Poly (dA:dT) and Poly (dG:dC)

**Figure 1:**
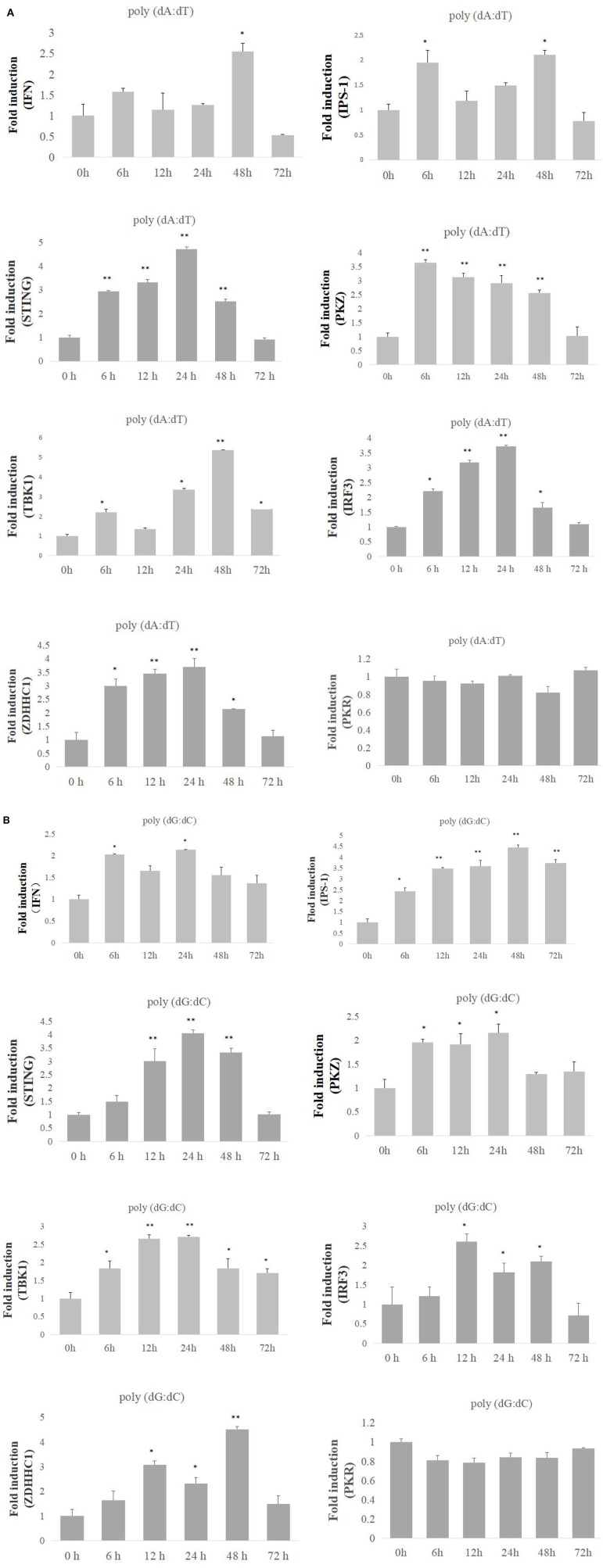
Expression profiles of PKZ and some other mediators of innate immune response. **(A,B)** CIK cells were seeded and incubated in 24-well plate for 12 h, and then the cells were separately transfected with poly (dA:dT) or poly (dG:dC) (2 μg/ml) for 0, 6, 12, 24, 48, and 72 h. Then the cells were sampled, and the mRNAs of IFN, IPS-1, STING, PKZ, TBK1, IRF3, and ZDHHC1 were detected by qRT-PCR. The mRNA expression levels of target genes were normalized relative to the reference gene β*-actin*. The groups of 0 h were controls, and the others (6, 12, 24, 48, 72 h) were experimental groups. Data represent mean ± SD (*n* = 3) of three experiments and were tested for statistical significance using unpaired two-tailed *t*-test ^*^p < 0.05, ^**^p < 0.01. The asterisk above the bracket indicated statistical significance between the control groups with experimental group.

To delineate the functional link of PKZ with IFN, IRF3, STING, ZDHHC1, IPS-1, and TBK1, their expression patterns in CIK cells were identified under the same conditions. *PKZ, IFN, IRF3, STING, and ZDHHC1* were upregulated under stimulation with poly (dA:dT) (Figure 1A) and poly (dG:dC) (Figure 1B) at mRNA level. It was also found that when the cells were stimulated with poly (dA:dT) and poly (dG:dC), *PKZ* transcripts showed a significant increase at the early phase of stimulation, and the transcripts of *STING, IRF3*, and *ZDHHC1* showed similar increases. The increase in transcripts of *IFN, IPS-1*, and *TBK1* appeared to be biphasic under stimulation with poly (dA: dT) rather than poly (dG: dC). PKR acted as a negative control in all cases. These results indicate that fish PKZ responds to poly (dA:dT) and poly (dG:dC) stimulations, just like some well-known mediators of immune response. PKZ is closely related to innate immune activity and may act as an up-stream protein under stimulation with poly (dA:dT) or poly (dG:dC).

### PKZ Is Located in the Cytoplasm

**Figure 2:**
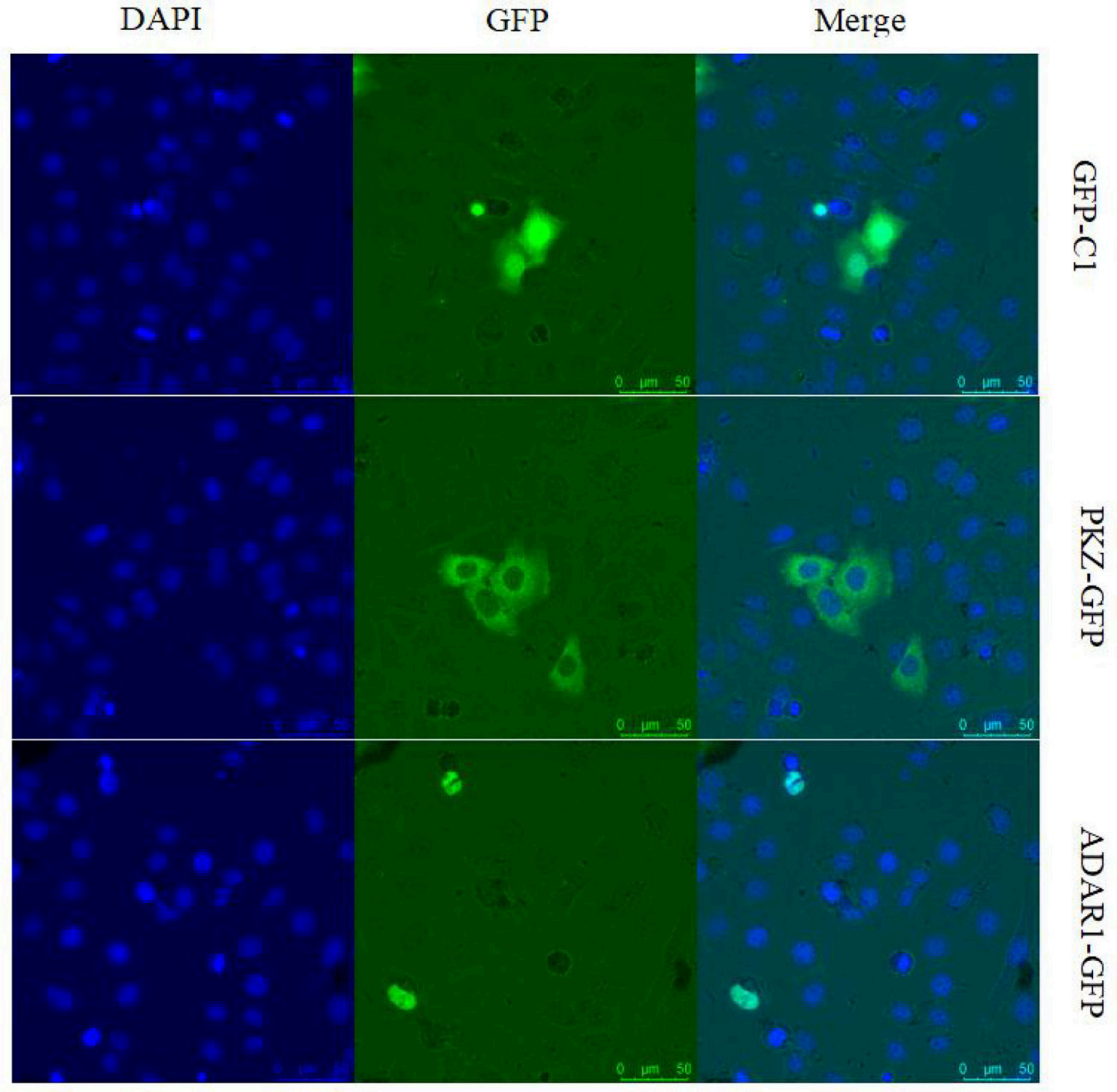
Subcellular localization analysis of grass carp PKZ. CO cells were seeded on 35 cm^2^ microscopy dishes and individually transfected with PKZ-pEGFP, ADAR1-pEGFP, and pEGFP-C1 plasmids. At 24 h post-transfection, the cells were fixed and examined using a confocal microscope. The scale bar is 25 μm. NA is 40× /0.60. This experiment is representative of at least three independent experiments.

It is necessary to determine the location of PKZ for its recognition of viral nucleic acids in fish cells. The plasmids PKZ-GFP and ADAR1-GFP were separately transfected into CO cells. Confocal microscopic detection suggests that PKZ-GFP is located in the cytoplasm; however, ADAR1-GFP (another Zα protein, used as a control) is located in the nuclear (Figure 2).

### PKZ Can Bind to Both Poly (dA:dT) and Poly (dG:dC) *in vitro*

**Figure 3:**
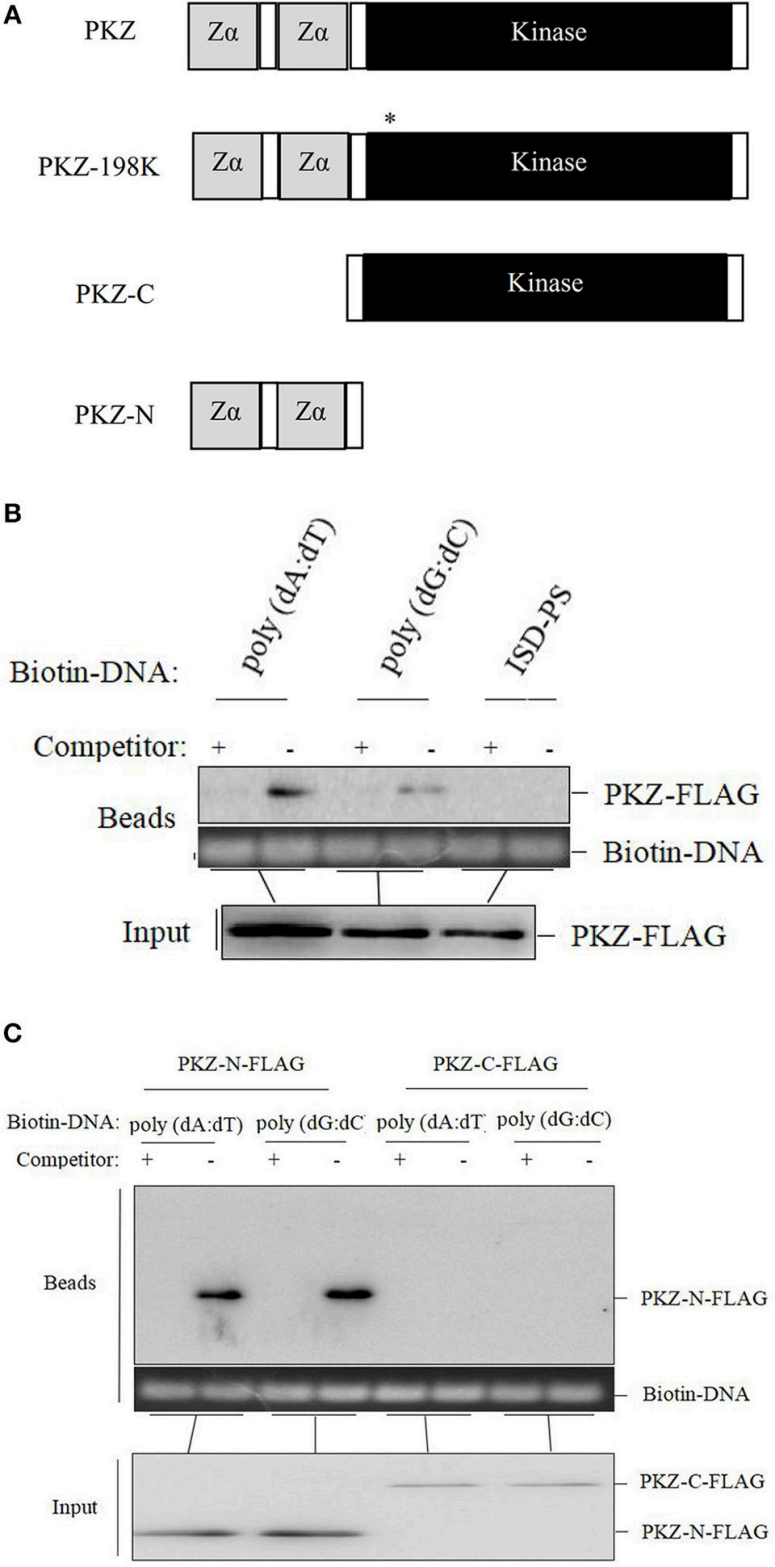
PKZ binds to poly (dA:dT) and poly (dG:dC) *in vitro*. **(A)** PKZ and the mutants. **(B)** CO cells were seeded on 10 cm diameter dishes and transfected with PKZ-FLAG, at 24 h post-transfection, cells were harvested, a total of 1% of PKZ-FLAG containing cell lysates was loaded as input, and the rest of lysates incubated with Dynabeads were separately immobilized with 2 μg of biotin-nucleic acids (poly (dA:dT), poly (dG:dC) and ISD-PS), and 10 μg of non-biotin tagged nucleic acids acted as competitors. The DNA in Dynabeads was detected in agarose gel. **(C)** PKZ mutants (PKZ-C-FLAG and PKZ-N-FLAG) were transfected into CO cells, at 24 h post-transfection, cells were harvested, a total of 1% of PKZ-FLAG containing cell lysates was loaded as input, and the rest of lysates incubated with Dynabeads were individually immobilized with biotin-nucleic acids (poly (dA:dT) and poly (dG:dC), and 10 μg of non-biotin tagged nucleic acids acted as competitors. The DNA in Dynabeads were examined in agarose gel. The molecular masses of PKZ-FLAG, PKZ-C-FLAG, and PKZ-N-FLAG are 67, 45, and 25 KD, respectively. Each Western blot is representative of at least three independent experiments.

PKZ has two Zα domains at its N-terminus and 11 conserved eIF2α kinase catalytic sub-domains at its C-terminus (Figure 3A). A series of PKZ protein mutants were constructed, including C-terminus of PKZ (*PKZ-C*-pcDNA3.1/FLAG), N-terminus of PKZ (*PKZ-N*-pcDNA3.1/FLAG), and Lys^198^ mutant of PKZ(*PKZ-198K*-pcDNA3.1/FLAG) and these were used for subsequent experiments (Figure 3A). It is known that the Lys^198^ mutant of PKZ lacks kinase activity (31, 33). To investigate the compatibility of PKZ with nucleic acids, nucleic acid pulldown assays were performed by incubating biotin-labeled DNA with PKZ protein. In this assay, non-biotin tagged nucleic acids [poly (dA:dT), poly (dG:dC), and ISD-PS] acted as competitors. High concentrations of non-biotin tagged nucleic acids in incubations (lysates and Dynabeads immobilized with biotin-nucleic acids) will result in competitive interaction with proteins. Qualitative analysis of nucleic acids-bound protein with ISD-PS being used as a negative control (Figure 3B) showed that PKZ-FLAG effectively binds to poly (dA:dT) and poly (dG:dC), but not to ISD-PS (RNA analog). While Zα domains (PKZ-N terminus) can combine with both poly (dA:dT) and poly (dG:dC), eIF2α kinase catalytic sub-domain (PKZ-C terminus) cannot with either of them (Figure 3C). These results indicate that fish PKZ can recognize and interact with DNA by its Zα domain.

### PKZ Is Activated by DNA Stimulation

**Figure 4:**
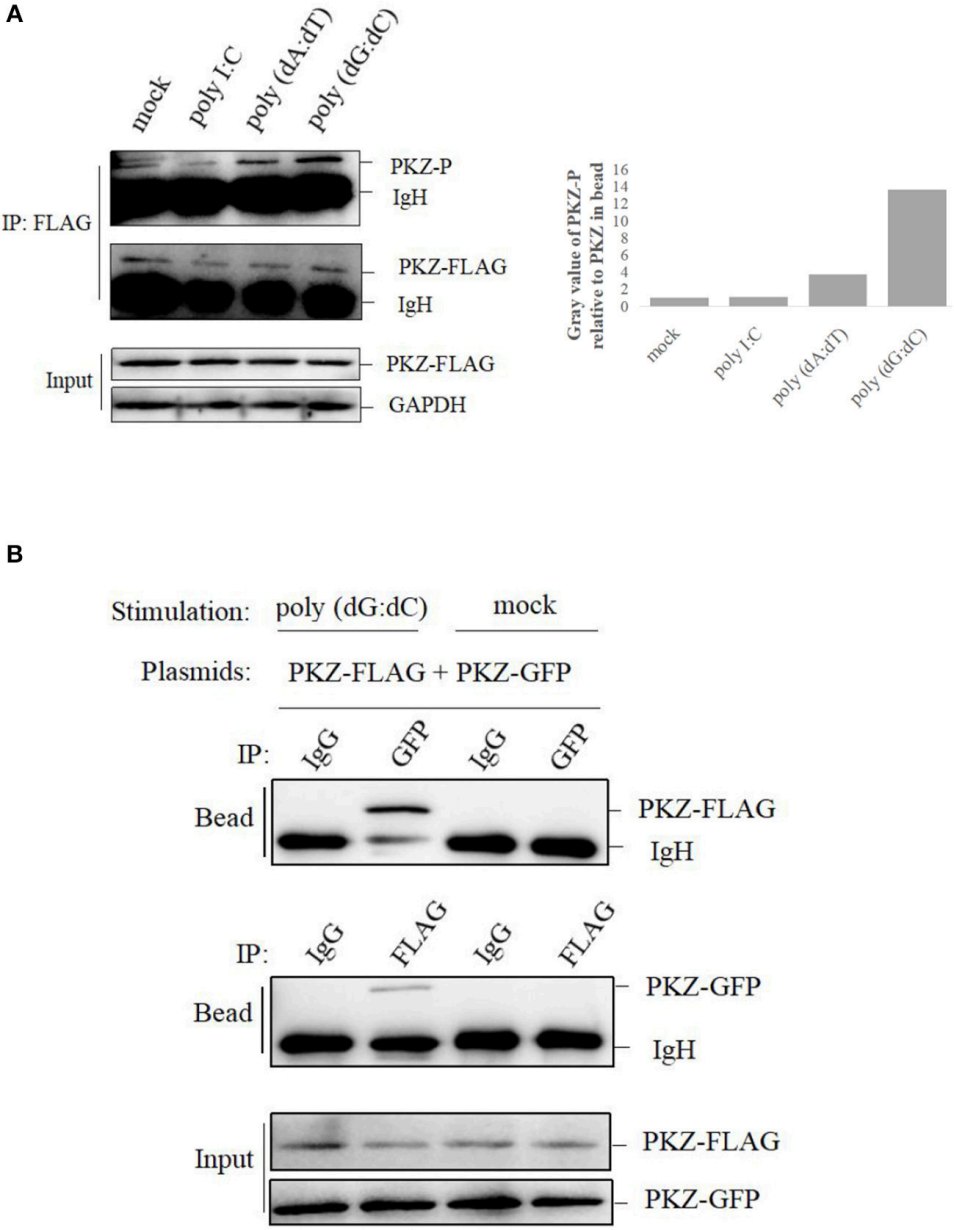
PKZ is activated by DNA-stimulation. **(A)** CO cells seeded in 10 cm diameter dishes were transfected with 2 μg PKZ-FLAG, 24 h later, negative control of cells was co-transfected with basic-pcDNA3.1, positive controls of cells were individually transfected with poly I:C, poly (dA:dT), poly (dG:dC), and experimental cells were co-transfected with PKZ-pcDNA3.1. After 12 h, cell lysates were immunoprecipitated with anti-FLAG antibody, then the immunoprecipitants were examined by Western blotting with anti-phospho-Ser/Thr/Tyr antibody (AnaSpec, CA). **(B)** Co-IP assays were carried out in HEK-293T cells. The cells seeded in 10 cm diameter dishes were separately co-transfected with PKZ-FLAG and PKZ-GFP. This experiment was divided into two groups: one group was stimulated with poly (dG:dC), and the other was not. Forty eight hours later, cell lysates were separately immunoprecipitated with anti-FLAG antibody, anti-GFP antibody and IgG (used as a control), then the immunoprecipitants were examined by Western blotting. Each Western blot is representative of at least three independent experiments.

To further investigate whether PKZ can be phosphorylated or not, CO cells were transfected with PKZ-FLAG. Twenty-four hours later, cells were separately transfected with 2 μg of poly I:C, 2 μg of poly (dA:dT), and 2 μg of poly (dG:dC) for 12 h. PKZ-FLAG protein was immunoprecipitated from cell lysates. The phosphorylation of PKZ-FLAG was detected with an anti-phospho-Ser/Thr/Tyr antibody. These results show that poly (dA:dT) and poly (dG:dC) can promote PKZ phosphorylation but poly I:C cannot; and poly (dG:dC) had stronger effects on PKZ than poly (dA:dT) (Figure 4A). Poly (dG:dC) was therefore selected as a stimulator for PKZ-dependent pathway in subsequent experiments.

Subsequently, two sets of PKZ constructs containing FLAG tag and GFP tag were used in the co-IP assay. Co-IP assay was performed for HEK-293T cells. The plasmids of PKZ-FLAG and PKZ-GFP were co-transfected into HEK-293T cells. Twenty-four hours post-transfection, the cells were divided into two groups, namely the poly (dG:dC)-stimulated group and a non-stimulated group. In the poly (dG:dC)-stimulated group, anti-GFP antibody-immunoprecipitated protein complex was detected by anti-FLAG (PKZ-FLAG) antibody; which was however not detected in the non-stimulated group. Likewise, anti-FLAG antibody-immunoprecipitated protein complex was detected with the anti-GFP (PKZ-GFP) antibody in the poly (dG:dC) stimulated group. IgG-immunoprecipitated protein complex was not detected with anti-GFP (PKZ-GFP) or anti-FLAG (PKZ-FLAG) antibody (Figure 4B). Co-IP results suggest that poly (dG:dC) promotes dimerization of PKZ. Overall, these findings demonstrate that fish PKZ can be activated by DNA stimulation.

### PKZ Initiates IFN Expression

**Figure 5:**
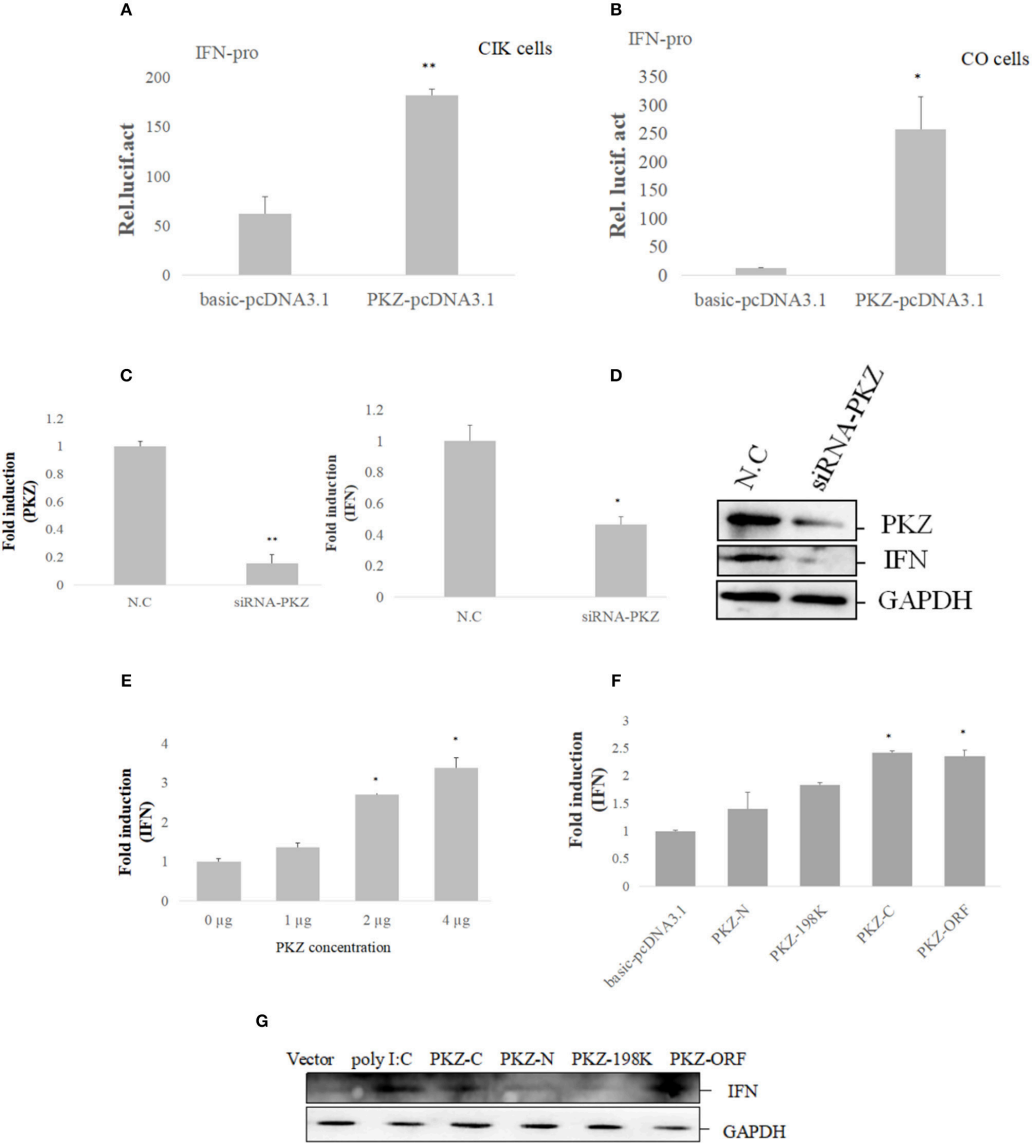
PKZ initiates IFN expression. **(A,B)** CIK and CO cells seeded in 24-well plates were co-transfected with IFN-pro and PKZ-pcDNA3.1. Basic-pcDNA3.1 was used as a control. A total of 0.025 μg of pRL-TK was included to standardize the expression level. Twenty four hours later, the cells were stimulated with poly (dG:dC) for 12 h. The transfected cells were harvested to detect the luciferase activities. Data were expressed mean ± SD (*n* = 3) and were tested for statistical significance using unpaired two-tailed *t*-test ^*^p < 0.05, ^**^p < 0.01. Statistical significance was analyzed between the control group (basic-pcDNA3.1) with experimental group (PKZ-pcDNA3.1). **(C,D)** CO cells seeded in 6-well plates were transfected with siRNA-PKZ. Twenty four hours later, the cells were stimulated with poly (dG:dC) for 12 h. The whole-cell mRNA and protein were prepared; qRT-PCR and Western blot were used to exam the expression profiles of PKZ and IFN when we knocked down PKZ in CO cells. The data from qRT-PCR represent mean ± SD (*n* = 3) and were tested for statistical significance using unpaired two-tailed *t*-test ^*^p < 0.05, ^**^p < 0.01. Statistical significance was analyzed between the control groups (N.C) and the experimental groups (siRNA-PKZ). **(E)** CO cells seeded in 6-well plates were individually transfected with PKZ-pcDNA3.1 in concentrations of 0, 1, 2, and 4 μg, then qRT-PCR was used to detect the expression of IFN. qRT-PCR represents mean ± SD (*n* = 3) and was tested for statistical significance using unpaired two-tailed *t*-test ^*^p < 0.05. Fold changes were determined relative to cell transfected with 0 μg of PKZ-pcDNA3.1. **(F,G)** CO cells seeded in 6-well plates were separately transfected with PKZ-pcDNA3.1, PKZ-C-pcDNA3.1, PKZ-N-pcDNA3.1, PKZ-198K-pcDNA3.1, and basic-pcDNA3.1. Twenty four hours later, the cells were stimulated with poly (dG:dC) for 12 h. qRT-PCR and Western blot were used to exam the induction of IFN. The data from qRT-PCR represent mean ± SD (*n* = 3) and were tested for statistical significance using unpaired two-tailed *t*-test ^*^p < 0.05. Statistical significance was analyzed between the control groups (basic-pcDNA3.1) and the experimental groups (PKZ-pcDNA3.1, PKZ-C-pcDNA3.1, PKZ-N-pcDNA3.1, PKZ-198K-pcDNA3.1). Each Western blot is representative of at least three independent experiments.

To confirm whether or not PKZ can trigger innate immunity, *PKZ*-pcDNA3.1 was over-expressed in both CIK cells (Figure 5A) and CO cells (Figure 5B). Twenty-four hours after transfection, cells were treated with equal amount of poly (dG:dC) for 6 h. The promoter activity of grass carp *IFN* was increasingly upregulated in comparison with that of the cells transfected with basic-pcDNA3.1, and results indicated that IFN transcription level was upregulated in CIK or CO cells overexpressed PKZ. However, after knockdown of PKZ, the mRNA and protein levels of PKZ and IFN were significantly downregulated (Figures 5C,D). In addition, *IFN* transcription level was upregulated by transfection with PKZ in higher concentrations in CO cells (stimulated with poly (dG:dC) of equal strength for 6 h prior to experiments) (Figure 5E). The transfection efficiency was shown in Supplemental Figure 1. These results indicate that PKZ plays critical roles in inducing IFN under DNA stimulation.

In subsequent assays, the mutants were used to investigate the relationship between the PKZ structure and IFN expression. The mRNA and protein levels of IFN were upregulated in the over-expressed PKZ-C terminus and PKZ-wt. However, no evidence was found that the PKZ-N terminus and Ser^198^ PKZ deficit contributed to IFN expression (Figures 5F,G). These data suggest that PKZ can promote IFN expression by PKZ-C terminus.

### PKZ Interacts With Mediators of IRF3- and ISGF3-Like Dependent Pathway

**Figure 6:**
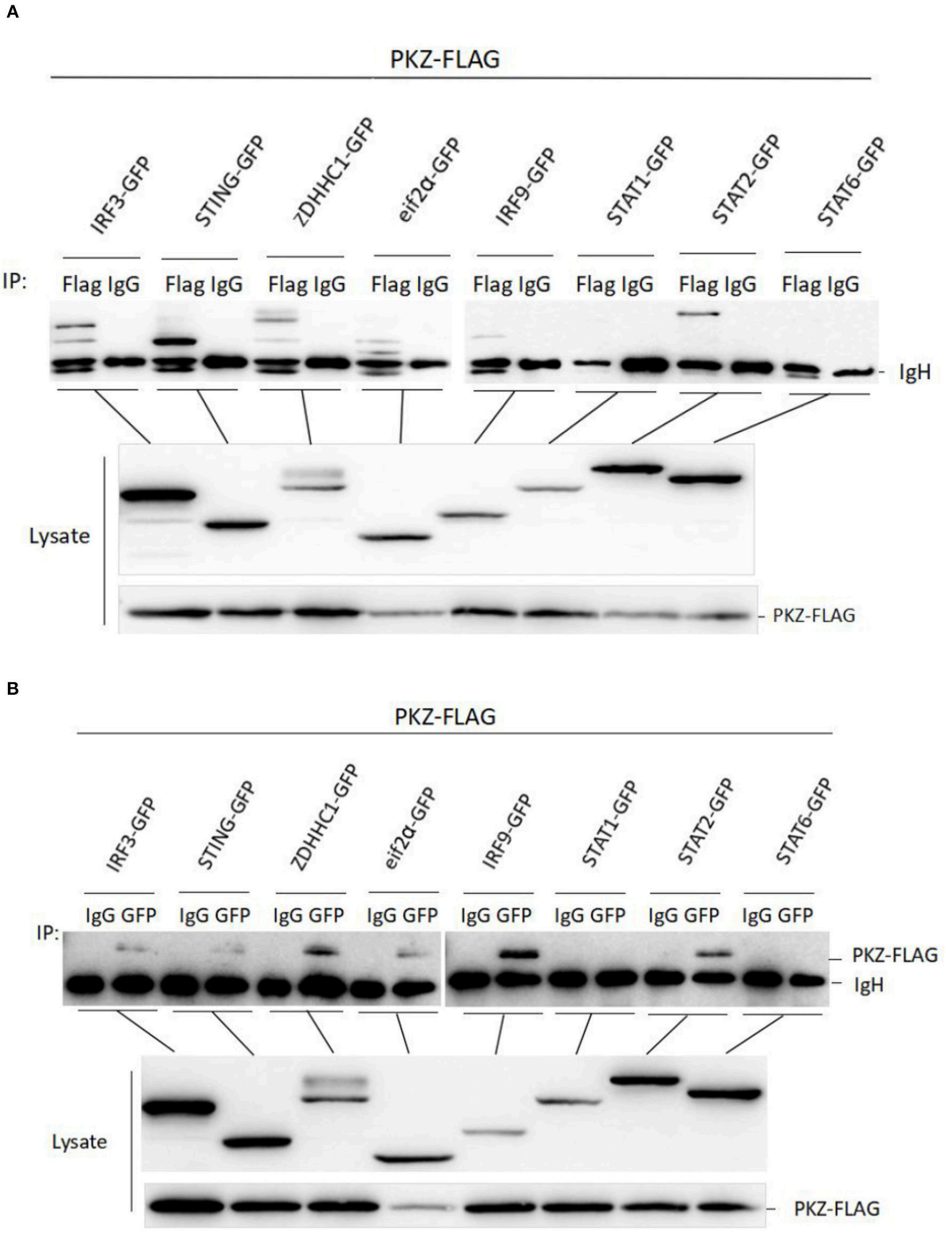
PKZ interacts with the mediators of IRF3-dependent and ISGF3-like dependent pathways. **(A,B)** HEK-293T cells seeded in 10 cm diameter dishes were separately co-transfected PKZ-FLAG with one of the following recombinant plasmids (IRF3-GFP, STING-GFP, ZDHHC1-GFP, eIF2α-GFP, IRF9-GFP, STAT1-GFP, STAT2-GFP, and STAT6-GFP), this experiment was divided into two groups: one group was immunoprecipitated with anti-FLAG antibody and the other group was immunoprecipitated with anti-GFP antibody. Forty eight hours later, cell lysates were separately immunoprecipitated with anti-FLAG (anti-GFP) antibody and IgG. Then the immunoprecipitants were examined by Western blot with the anti-GFP (anti-FLAG) antibody. The molecular masses of IRF3-GFP, STING-GFP, ZDHHC1-GFP, eIF2α-GFP, IRF9-GFP, STAT1-GFP, STAT2-GFP, and STAT6-GFP are 85, 70, 85, 60, 80, 90, 98, and 87 kD, respectively. Each Western blot is representative of at least three independent experiments.

To further explore the mechanism of PKZ upregulating IFN expression, it is necessary to identify the PKZ-dependent pathway. A previous study has found that PKZ can interact with eIF2α (31), so eIF2α was chosen as a positive control in this experiment. Co-IP assays were performed with HEK293T cells, in which *PKZ*-FLAG were individually co-overexpressed with the following recombinant plasmids (*IRF3*-GFP, *STING*-GFP, *ZDHHC1*-GFP, *IRF9*-GFP, *STAT1*-GFP, *STAT2*-GFP, *STAT6*-GFP, and *eIF2*α-GFP). Forty-eight hours post-transfection, the cells were stimulated with poly (dG:dC) for 6 h before all the proteins were harvested. IgG-immunoprecipitation acts as a negative control, anti-FLAG antibody-immunoprecipitation (Figure 6A) and anti-GFP antibody-immunoprecipitation (Figure 6B) show significant interaction between PKZ-FLAG and IRF3-GFP, STING-GFP, ZDHHC1-GFP, eIF2α-GFP, IRF9-GFP, and STAT2-GFP. These results show that PKZ can interact with IRF3, STING, ZDHHC1, eIF2α, IRF9, and STAT2.

### PKZ Initiates IFN Expression Through IRF3- and ISGF3-Like Dependent Pathways

**Figure 7:**
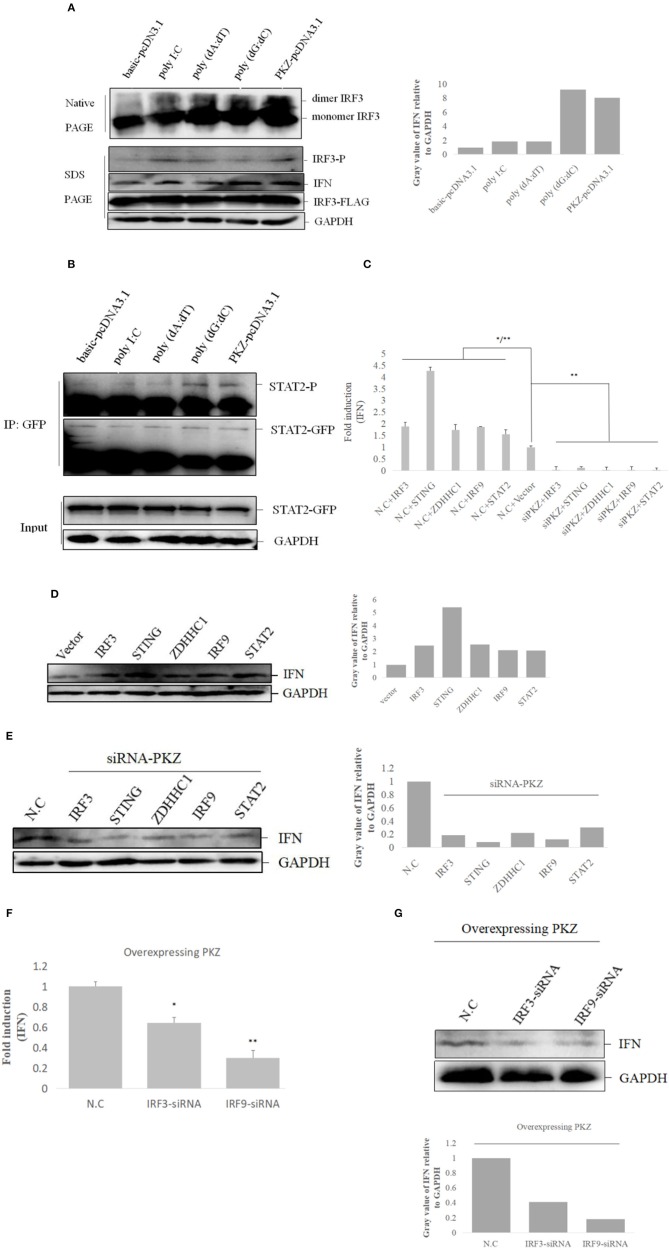
PKZ initiates IFN expression via IRF3-dependent and ISGF3-like-dependent pathways**. (A)** CO cells seeded in 10 cm diameter dishes were separately transfected with the negative control (basic-pcDNA3.1), the positive control [poly I:C, poly (dA:dT), poly (dG:dC)] and the experimental group (PKZ-pcDNA3.1) plasmids. Twenty four hours after transfection, the transfected cells were harvested for SDS-PAGE or native PAGE and immunoblotting with the indicated antibodies. **(B)** CO cells seeded in 10 cm diameter dishes were transfected with STAT2-GFP. Twenty four hours after transfection, the cells were separately transfected with the negative control (basic-pcDNA3.1), the positive controls [poly I:C, poly (dA:dT), poly (dG:dC)], and the experimental group (PKZ-pcDNA3.1) plasmids for 12 h. The cell lysates were immunoprecipitated with anti-GFP antibody, the immunoprecipitants were examined by Western blot with anti-phospho-Ser/Thr/Tyr antibody (AnaSpec, CA). **(C–E)** CO cells seeded in 24-well plates were transfected with siRNA-PKZ or N.C. Twenty four hours after transfection, the cells were separately transfected with IRF3-pcDNA3.1, STING-pcDNA3.1, ZDHHC1-pcDNA3.1, IRF9-pcDNA3.1, STAT2-pcDNA3.1, and basic-pcDNA3.1 in siRNA-PKZ groups and N.C. groups. The cells were harvested to carry out qRT-PCR and Western blotting. The data from qRT-PCR represent mean ± SD (*n* = 3) and were tested for statistical significance using unpaired two-tailed *t*-test ^*^p < 0.05, ^**^p < 0.01. The asterisk above the error bars indicated statistical significance using the group co-transfected with basic-pcDNA3.1 and N.C. The asterisk above the bracket indicated statistical significance between the groups transfected N.C and the groups transfected with PKZ-siRNA **(F,G**). The whole-cell mRNA or protein were prepared, qRT-PCR and Western blot were used to exam the expression of IFN when we knocked down IRF3 or IRF9 in CO cells. Endogenous GAPDH was detected as a loading control. The data from qRT-PCR represent mean ± SD (*n* = 3) and were tested for statistical significance using unpaired two-tailed *t*-test. Asterisks indicate significant differences from control (N.C) ^*^p < 0.05, ^**^p < 0.01. Fold changes were determined relative to cell transfected with N.C. Each Western blot is representative of at least three independent experiments.

**Figure 8:**
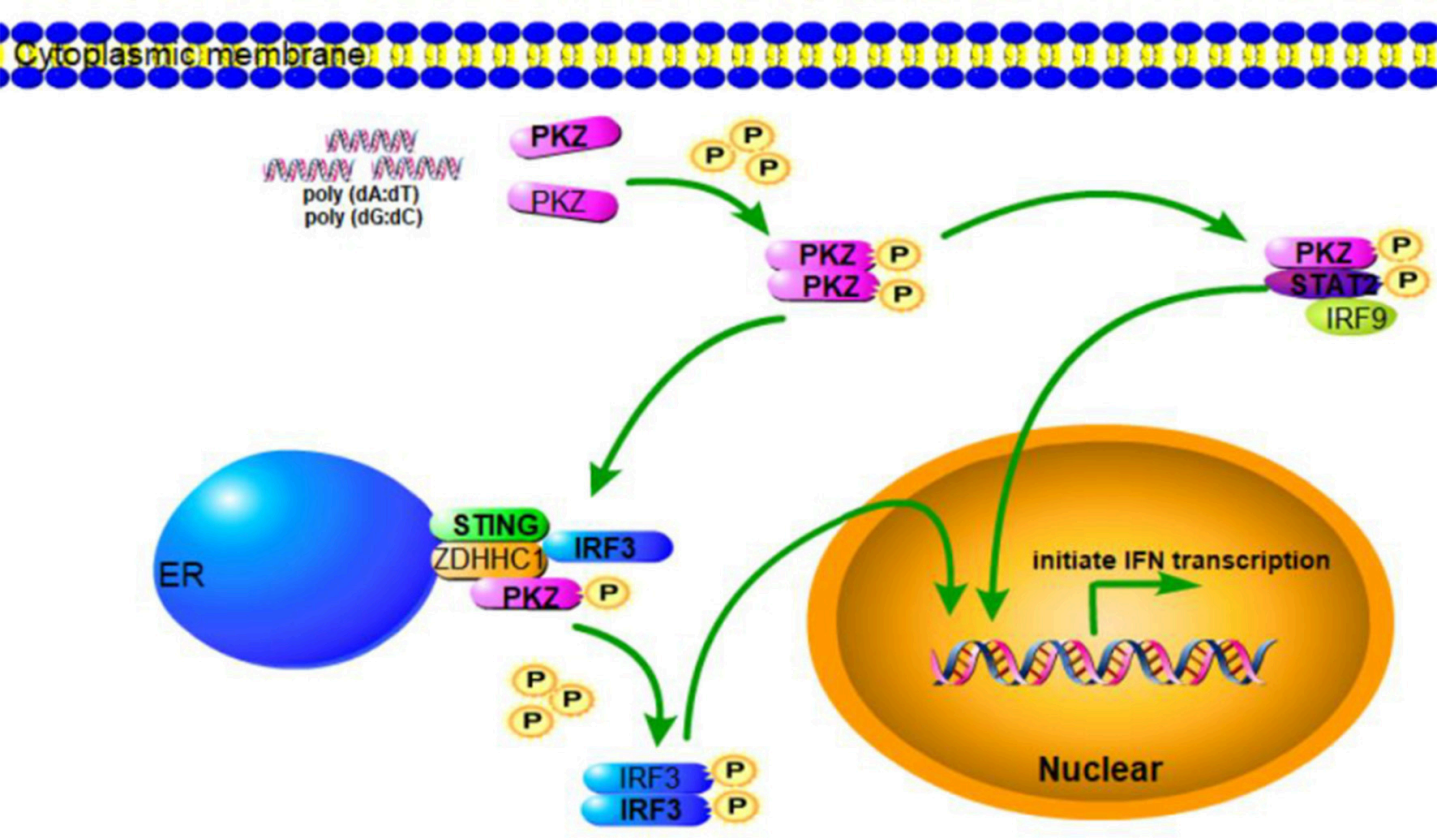
Schematic representation of PKZ-mediated signaling pathway. In response to DNA infection, PKZ-mediated IFN signaling pathway is activated. PKZ interacts with the mediators of IRF3-dependent and ISGF3-like dependent signaling pathways and phosphorylates IRF3 and STAT2, and thus helps induce immune responses.

Subsequent experiments were conducted to determine the relationships among PKZ and some common mediators of innate immunity. Dimerization and phosphorylation levels of IRF3 were increased when CO cells were transfected with poly I:C, poly (dA:dT), poly (dG:dC) and *PKZ*-pcDNA3.1, with pcDNA3.1-basic acting as negative control (Figure 7A). Interestingly, STAT2 phosphorylation activity was also enhanced in the process (Figure 7B). Moreover, IFN expression was upregulated in CO cells in which IRF3, STING, ZDHHC1, IRF9, and STAT2 were overexpressed. However, the knockdown of PKZ blocked the IFN expression (Figures 7C–E), whereas knockdown of IRF3 or IRF9 inhibited the IFN expression through PKZ (Figures 7F,G). The effect of *siRNA-IRF9* was examined in Supplemental Figure 2. These data suggest that PKZ initiated immune responses through mediators of the IRF3 dependent pathway (IRF3, STING, and ZDHHC1) as well as the ISGF3-like (IRF9 and STAT2) dependent pathway (Figure 8).

## Discussion

In mammals, eIF2α is phosphorylated with one of the eIF2α kinases that consist of PKR, PKR-like ER kinase (PERK), general control non-derepressible-2 (GCN2), and heme-regulated eIF2α kinase (HRI) (38). Among these kinases, PKR is most widely studied. PKR is known to mediate IκB-kinase β (IKKβ) phosphorylation and be able to activate the NF-κB pathway (39). PKR also inhibits viral protein synthesis via eIF2α phosphorylation (40) and thus helps resist virus infection (41, 42). Because the fish-specific protein kinase PKZ is homologous with mammalian PKR, it was believed—for a short while—that it is a duplicate of mammalian PKR (28–30). That PKZ possesses some functions similar to those of PKR (31, 33, 43–45) is not in question.

Fish PKZ have been identified in *C. idellus, Carassius auratus, Atlantic salmon, Gobiocypris rarus*, and *Danio rerio* (28–30, 32, 44). BLAST homologous research indicates that *C. idellus* PKZ full-length cDNA has 2,158 bp with a largest open reading frame (ORF) encoding 513 amino acid and shares high-level homology with other fish PKZ (32). All fish PKZ contain two Zα domain in N-terminus and a conserved catalytic domain in C-terminus (28–30, 32, 44).

*Ctenopharyngodon idellus* PKZ induces the apoptosis through eIF2α phosphorylation (33). In the same way dsRNA induction lead to PKR auto-phosphorylation and auto-dimerization (46–48), PKZ can interact with the [poly (dA:dT) DNA or poly (dG:dC)], though it cannot interact with dsRNA (Figure 3), just as poly (dA:dT) and poly (dG:dC) can facilitate PKZ phosphorylation but poly I:C cannot (Figure 4A). Because of this, poly (dG:dC) was chosen as activator for PKZ in our subsequent study. Besides PKZ phosphorylation, poly (dG:dC) promotes PKZ dimerization (Figure 4B). Consistently with our results, the N-terminus of crucian carp PKZ was found not to interact with poly I:C and this indicates that dsRNA is unable to activate PKZ (31). It was also observed that PKZ is located in the cytoplasm (Figure 2), where the binding with infected DNA takes place. We believe strongly that PKZ may act as an essential cellular DNA receptor.

It is well-known that DNA sensors can promote IFN-β expression, and PKZ facilitates type I IFN expression in different grass carp cell lines, which indicates how so that PKZ has identical functions in various types of fish cells (Figures 5A–D). As indicated, IFN expression was gradually upregulated with increasing concentrations of overexpressed PKZ in the experiments (Figure 5E), and there is clear evidence of functional differentiation in the kinase domain and Zα domain of PKZ. The N-terminal Zα domain is mainly responsible for recognizing cytoplasmic DNA and also has B-Z transition activity (49, 50). The C-terminal kinase catalytic domain of PKZ plays a significant role in triggering IFN expression (Figures 5F,G). A similar observation was also documented in cGAS (16). Liu et al. (31) have also suggested that fish PKZ is a new cytosolic sensor for DNA detection by virtue of the unique N-terminal Zα domains.

DNA-sensed PRRs used to make intrinsic immune-stimulating properties of plasmid DNA vaccines can recognize intracellular DNA (51). For example, DAI can be used to make intrinsic immune-stimulating property of plasmid DNA vaccines which promote the transcription of genes encoding type I IFNs, proinflammatory cytokines, and co-stimulatory molecules (52). Our findings may provide a potential applicability for DNA vaccines. This is to say that PKZ also could be used to make DNA vaccines. Meanwhile, PKZ provides mediated basics for poly (dA:dT) and poly (dG:dC) acting as immune inducers in up-regulation of IFN. We believe that PKZ could be used to make DNA vaccines and our findings may be a useful contribution in that regard.

Though PKZ is known to be able to initiate IFN expression, the PKZ-mediated pathways need further study, which was done in our subsequent experiments. The IFN-induction pathway mainly includes IRF3-dependent and ISGF3-dependent pathways (53, 54). The IRF3-dependent pathway is primarily responsible for IFN and ISGs expression at the early stages of viral infection (55), while the ISGF3-dependent pathway is responsible at the later period of infection (56). It is well-known that IRF3 (35), STING (21), and ZDHHC1 (57) are the members of the IRF3-dependent pathway. Our previous results show that grass carp IRF3, STING, and ZDHHC1 are all upregulated under stimulation with poly (dA:dT) and poly (dG:dC) (35). The expression characteristic of PKZ is similar to those of IRF3, STING, and ZDHHC1 under stimulations with different DNAs (Figure 1). In addition, PKZ can directly interact with these mediators under stimulation by poly (dG:dC) (Figure 6).

ISGF3 (IRF9/STAT1/STAT2) acts as a transcription regulator induced by IFN (58). However, many studies have shown that STAT2 rather than STAT1 is the catalytic domain for ISGF3 (59, 60). Therefore, IRF9 and STAT2 are the major subunits of the ISGF3 complex. This functional unit is known as ISGF3-like. Our previous studies also found that grass carp IRF9 and STAT2 can form an activated heterodimer (61). In this paper, we have shown that PKZ can interact directly with ISGF3-like under stimulation with poly (dG:dC) (Figure 6). We believe it likely that PKZ triggers antiviral activity in an ISGF3-like dependent pathway.

PKZ was shown to promote the IRF3 dimerization and phosphorylation (Figure 7A) and increase STAT2 phosphorylation (Figure 7B). These results indicate that PKZ can respond to DNA stimulation and activate IRF3 and ISGF3-like. IFN expression is activated in IRF3- and ISGF3-like-mediated pathways, which are dependent on the presence of PKZ (Figures 7C–E).

In conclusion, when cells are invaded by pathogenic DNA, PKZ recognizes it and then binds with it. The activated PKZ first interacts with and activates the mediators of the IRF3-dependent pathway to form the tetramer of PKZ-IRF3-STING-ZDHHC1, and then activates IRF3. In the ISGF3-like mediated pathway, the activated PKZ can activate the dimer of IRF9-STAT2 to form the trimer of PKZ-IRF9-STAT2 (Figure 8). Notably, these data indicate that fish PKZ gives antiviral signals in IRF3-dependent or ISGF3-like dependent pathways under stimulation with DNA, and thus helps initiate immune responses.

## Author Contributions

CH supervised the research. XX conceived the study, designed, and performed the experiments and wrote the manuscript. ML, XX, DL, BC, HM, and CW analyzed the experiments and data. ZJ, and CL provided reagents, technical assistance and contributed to completion of the study. DL revised the manuscript. All authors reviewed the results and approved the final version of the manuscript.

### Conflict of Interest Statement

The authors declare that the research was conducted in the absence of any commercial or financial relationships that could be construed as a potential conflict of interest.
