# Supplementary figures and images for: The Fish-Specific Protein Kinase (PKZ) Initiates Innate Immune Responses via IRF3- and ISGF3-Like Mediated Pathways
Source: Front Immunol. 2019 Mar 28;10:582. doi: 10.3389/fimmu.2019.00582 (PMC6447671; doi:10.3389/fimmu.2019.00582)

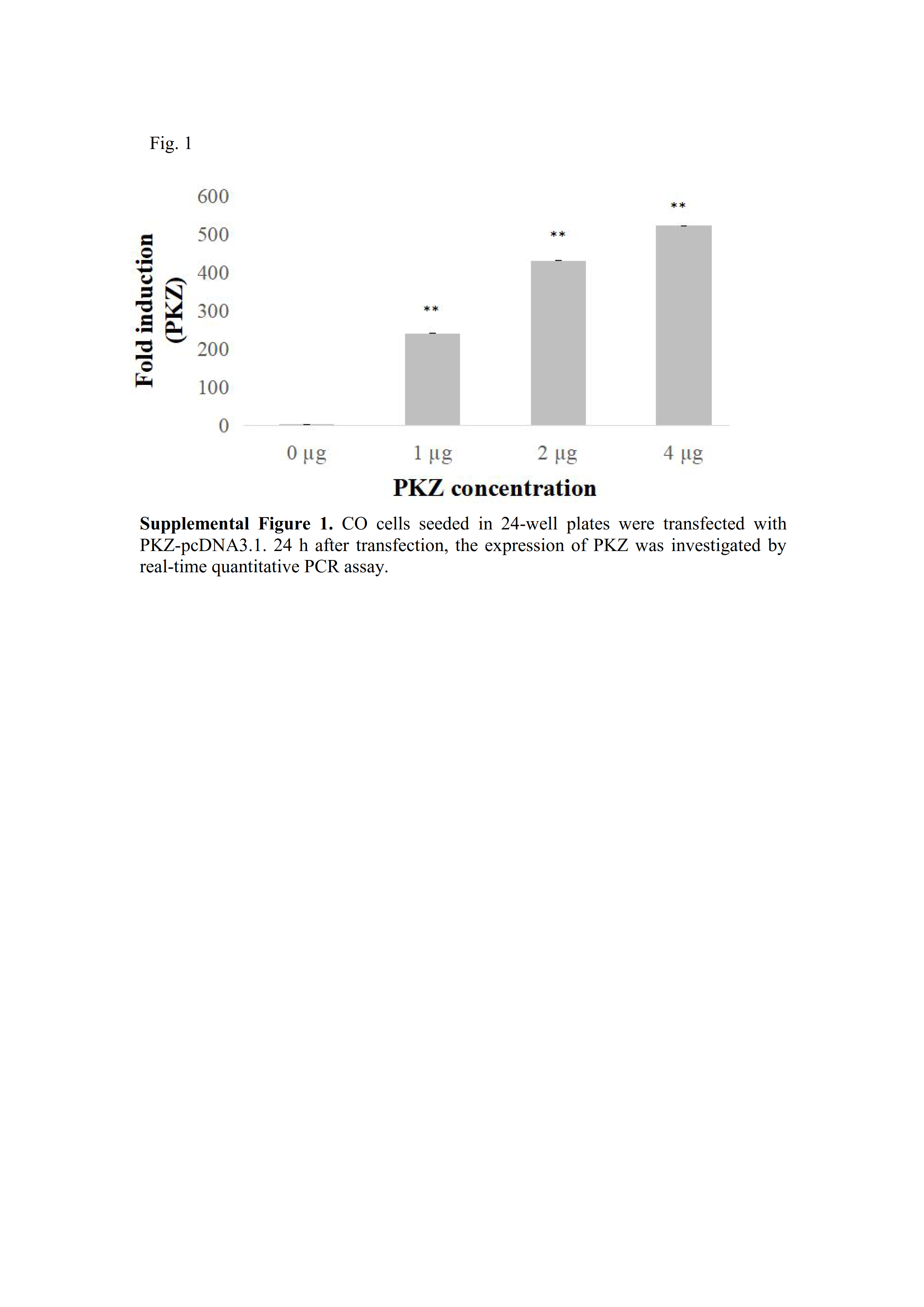

Supplement: Supplementary file 1 [file Image_1.TIF]

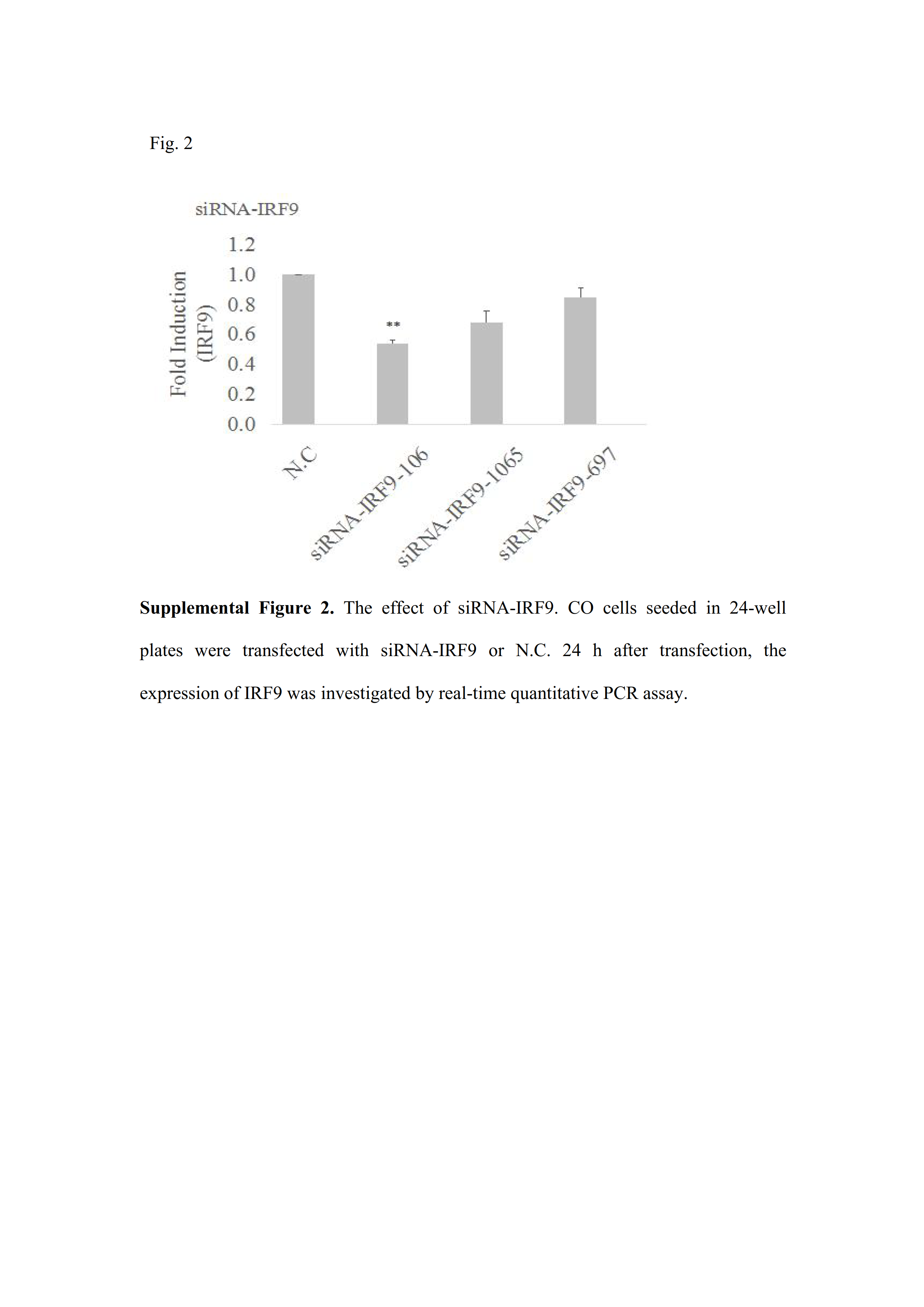

Supplement: Supplementary file 2 [file Image_2.TIF]
